# Supplementary material for: The vast majority of somatic mutations in plants are layer-specific
Source: Genome Biol. 2024 Jul 24;25:194. doi: 10.1186/s13059-024-03337-0 (PMC11267851; doi:10.1186/s13059-024-03337-0)
Supplement: Supplementary file 3 — Additional file 3. Review history. [file 13059_2024_3337_MOESM3_ESM.docx]

Review History

**First round of review**

**Reviewer 1**

**Were you able to assess all statistics in the manuscript, including the appropriateness of statistical tests used?**

Yes, and I have assessed the statistics in my report.

**Comments to author:**

Summary:
Goel and colleagues present a compelling investigation of tissue-specific sequence-level mutations in plants. Using deep sequencing in leaves and specific layers of fruits, the study reveals that the L1 layer exhibits a higher rate of mutation accumulation compared to L2, and that fruits tend to inherit mutations from the L2 layer. Another significant observation is that mutations occur less frequently in coding regions. By comparing mutations between the L1 and L2 layers, the study confirms the distinct developmental histories for these layers, with L1 cells sharing more mutations across branches than with L2 cells of the same branch. With single-cell RNAseq they confirm that layer-specific mutations affect transcripts in a layer-specific manner. This paper contributes valuable insights into the field of plant development and genomic mutation and is likely to be of broad interest. The paper is well-written and the conclusions are well-supported by the data. The scientific rigor and merit are commendable and leave no major concerns - the minor comments below are thoughts and suggestions that I leave to the authors/editors to decide what is essential for publication.

Minor comments:

Line 228: "Meristematic mutations affect the expression of genes only in the respective layer"... should this more accurately read "Meristematic mutations affect the transcripts of genes only in the respective layer" (expression > transcripts). It wasnt clear that the mutations alter expression level, which may be how readers will interpret the original phrasing.

In my opinion, Goel et al's investigation is significantly more robust and compelling than another recent and related study in the field (Schimtt et al. 2024, PNAS, https://www.pnas.org/doi/abs/10.1073/pnas.2313312121). The authors should cite that work and consider discussing.

Mutation calling comments
The author's variant calling approach appears robust. However, given the depth of sequencing is quite high, I wonder if they considered Strelka2, Octopus, Mutect or some other variant caller optimized for such data to identify/validate somatic mutations? Table S1 indicates variability among samples for coverage. Did the authors consider controlling for depth of L1/L2/Leaf samples? And for calling true positives, would it be a good idea to use % depth rather than total read support (i.e. 50/400 reads is different than 50/200 reads)?

I wonder about the consequence of variant calling pipeline for intragenome mutation rate heterogeneity. For example, is the denominator of Fig. 1G be affected by regions of the genome that are uncallable (e.g. due to low mappability, etc)? It might be worth ensuring that uncallable regions are not included in the estimate of total genome space for each class of genomic region. Tools like Strelka2 provide a list of callable regions to achieve this.

L119: Leaf only mutations: might these be L3?

Line 175-178: Any more info on mother tree or when this cross might have taken place and how many rounds of clonal propagation since?

Additional discussion on various results may be useful to readers and increase the impact of the paper.
Line 129: Higher L1 mutation rate. This result is particularly compelling. Perhaps since L1 doesnt contribute to germline, there has been relaxed selection (at long-term evolutionary timescales) on mechanisms promoting DNA repair in L1? Have the authors considered testing for differences in the expression of DNA repair genes between cell layer types using their single-cell RNAseq datasets? It might be worth speculating on consequences for clonal plants/crops/breeding, such as the transgenerational stability of somatic "sports" depending on the cell layer?
.
Line 162: L1 are more similar between branches than with L2 of same branch. This is a main point. Do the authors mean to reference Figure 1J to show this? I wonder if a version of Fig S10 which combines L1 and L2 into a single distance tree would be valuable?

L 343: "genome sequencing (scDNA-seq)" is mentioned but it wasnt immediately clear where scDNA-seq appears in results?

**Reviewer 2**

**Were you able to assess all statistics in the manuscript, including the appropriateness of statistical tests used?**

There are no statistics in the manuscript.

**Comments to author:**

This manuscript describes the distribution of somatic mutations associated with the L1 and L2 lineages in apricot using tissue specific DNA sampling and single-cell RNA sampling. The authors state that they can disregard the contribution of the L3 layer. They report a high number of accumulated mutations in both the L1 and L2 layers, shared across seven fruit sampled from seven major branch units across the tree. This is one of the first studies to assign mutations to specific cell layers in plants. Moreover, it uses two RNA methodologies scRNA seq and SclSO-seq that are among the first applications of such methods to somatic mutation studies. While the study is novel there are also several criticisms that relate to inference regarding the layers of the fruit samples.

Most important of these is the author statement (Lines 101-2) that they can disregard the contribution of the L3 layer. Derman (1956) seems to show clearly through the cytochimeric mutants (Fig. 13) and with the L3 bud sports that the L3 layer is fully intercalated with L2 in the mesocarp of the fruit of peach, which is a closely allied crop. Thus, isn't it possible that some of the SMs in the current study that are classed as L2 mutations could possibly be L3 mutations? Given that all progeny from this tree will all derived from L2, as stated by the authors, then the answer could be determined from the progeny or successive branches in L2 of next years. Alternatively, if progeny data are not available, then the authors could add language to leave the interpretation more open to a contribution of L3. Given that Stewart and Dermen (1975) suggest that L3 is often replaced by L2 along shoots, this could be mentioned to support the emphasis on L2.

The other sampling issue relates to the suture line of the fruit which is L1 only, of two cell thickness, and entirely bissects the fruit interior. This presence of L1 in the interior of the fruit is discussed in several figures in the Derman (1956) paper, notably Fig. 15 and Fig. 6D. It was unclear to this reviewer how the mesocarp sampling in the current manuscript addressed the suture region. Was effort made to avoid the suture line and thus possible presence of L1 cells into the collected samples? The reader could not find text addressing this issue in the methods section. Currently, the manuscript reports that twelve mutations are shared by L1 and L2 (Fig. 2B and Fig. 1J far right bar group) and the text interprets that this could be due to layer replacement (Lines 124-5). If layer replacement were actually to occur in the meristem leading to that fruit,would it not have a much more profound effect on the mutation distribution of the whole distal samples? Thus, to this reader, the more likely conclusion is a small amount of L1 cell contamination into the L2 samples from the suture zone.

Figure 3 needs more guidance for the reader. In panel 3B, does Cluster 0 represent the largest group of cells and Cluster 14 represent the smallest group of cells? The label for Cluster six is possibly missing. For panel 3C, the reader is shown the observed hits of cells with each of the six mutations, but the reader cannot tell from the panel or the legend what was expected. Were these six mutations supposed to be present in all four branches? If some of them were only expected in a subset of branches, then it will help to have that information, at least in the figure legend.

Additional comments:
1. Legend for Fig. 1C could use clarification of the units of the Y-axis.
2. Fig. 2C, the number of SMs that were used to determine the percentages shown would help. Not clear to the reader how the two panels differ and how data from specific mutations were used. Are data from all of the mutations shown here or just for a subset of mutations?
3. What is the cutoff in Appendix Table S4 for allele to be considered present in a sample? Not clear to the reader how to understand the quantitative variation shown there.

**Reviewer 3**

**Were you able to assess all statistics in the manuscript, including the appropriateness of statistical tests used?**

No, I do not feel adequately qualified to assess the statistics.

**Comments to author:**

This manuscript studied somatic mutations in distinct cell layers of fruit samples collected from multiple branches of an Apricot tree. The results showed that >90% of the somatic mutations were specific to individual layers. Overall, the findings of this study are generally interesting, and important for plant breeding research as well.
Although the study is well done, I have a few questions or suggestions.
1) To me, it is difficult to imagine the different layers of the tree across a big tree evolved like this study: different layer has different mutation rate, and the same layer share more mutations among different branches. It is important to give some explanation based on the references or proposal some assumptions about this.
2) Initially, when I read the manuscript, I assumed different branches mimics mutation accumulation lines, it will be important to discuss the similarity and difference between MA study and this study.
3) The reference genome used here are assembled with the reads produced from leaves of the seven different branches. I would assume the assembled genome must already contain most of the mutation of different branches, which is kindly a mixed genome. To what extent this mixed genome could affect variation identification and final results?

**Authors Response**

**Point-by-point responses to the reviewers’ comments:**

# Reviewer #1

Goel and colleagues present a compelling investigation of tissue-specific sequence-level mutations in plants. Using deep sequencing in leaves and specific layers of fruits, the study reveals that the L1 layer exhibits a higher rate of mutation accumulation compared to L2, and that fruits tend to inherit mutations from the L2 layer. Another significant observation is that mutations occur less frequently in coding regions. By comparing mutations between the L1 and L2 layers, the study confirms the distinct developmental histories for these layers, with L1 cells sharing more mutations across branches than with L2 cells of the same branch. With single-cell RNAseq they confirm that layer-specific mutations affect transcripts in a layerspecific manner. This paper contributes valuable insights into the field of plant development and genomic mutation and is likely to be of broad interest. The paper is well-written and the conclusions are well-supported by the data. The scientific rigor and merit are commendable and leave no major concerns - the minor comments below are thoughts and suggestions that I leave to the authors/editors to decide what is essential for publication.

*Response: Thank you very much for this very kind and supportive evaluation. This highlights the important findings of our study and that they are well-supported by the data presented.*

**Minor comments:**

Line 228: "Meristematic mutations affect the expression of genes only in the respective layer"... should this more accurately read "Meristematic mutations affect the transcripts of genes only in the respective layer" (expression > transcripts). It wasnt clear that the mutations alter expression level, which may be how readers will interpret the original phrasing.

*Response: Agreed, we have changed the line accordingly.*

In my opinion, Goel et al's investigation is significantly more robust and compelling than another recent and related study in the field (Schimtt et al. 2024, PNAS, https://www.pnas.org/doi/abs/10.1073/pnas.2313312121). The authors should cite that work and consider discussing.

*Response: Thanks for the suggestion, we now discuss this study in the Conclusion section.*

Mutation calling comments

The author's variant calling approach appears robust. However, given the depth of sequencing is quite high, I wonder if they considered Strelka2, Octopus, Mutect or some other variant caller optimized for such data to identify/validate somatic mutations?

*Response: Current methods for somatic mutation identification (like Strelka2, Mutect2 etc) are targeted towards cancer genomics, where somatic mutations are searched in paired tumour and normal samples. Typically, mutations present in the tumour samples but absent in the normal (background) samples are identified as somatic mutations. For somatic mutations in trees, more sophisticated background correction methods are required because they can be shared between different layers and different branches.*

*Nevertheless, we tested somatic mutation identification using Strelka2 and Mutect2. Here, we considered one sample as the “tumour” and the others as the background samples. However, manual checking of the (many) somatic mutations that were called by these tools showed inconsistent results, which pointed towards the presence of false positive calls (as also previously shown by Wang et al. (https://doi.org/10.1186/s13059-021-02285-3)). For example, a preliminary comparison between two selected branches, identified more than 2,100 indels in one of the branches, most of which were false positives according to manual revision of the read alignments. Therefore, we developed custom pipelines that were better suited for somatic mutation calling in our dataset.*

Table S1 indicates variability among samples for coverage. Did the authors consider controlling for depth of L1/L2/Leaf samples? And for calling true positives, would it be a good idea to use % depth rather than total read support (i.e. 50/400 reads is different than 50/200 reads)?

*Response: We agree, and in fact, we have applied sample (i.e., coverage) specific thresholds. Our pipeline uses sample-specific lower and upper bounds for read depth in addition to % depth. Table S1 reports the coverage estimated from raw fastq files. The raw reads were aligned to the reference genome, followed by filtering of duplicated reads. We then get read depth at each genomic position consisting of reads with high mapping quality (>=10) and highly accurate base quality (>=30). We used the distribution of these read depths to select sample-specific lower and upper bounds for read depths. These are reported in Table S2.*

*We have updated the Methods section to explain this better and added a new Supplementary Figure S4 that visualises the filtered read depth distributions.*

I wonder about the consequence of variant calling pipeline for intragenome mutation rate heterogeneity. For example, is the denominator of Fig. 1G be affected by regions of the genome that are uncallable (e.g. due to low mappability, etc)? It might be worth ensuring that uncallable regions are not included in the estimate of total genome space for each class of genomic region. Tools like Strelka2 provide a list of callable regions to achieve this.

*Response: Thank you for this helpful suggestion. Now, we filtered out uncallable regions (regions where few reads mapped with high mapping and base quality) from the analysis and have updated Figure 1G. We also re-assessed if the presence/absence of mutations in specific regions is significantly different from a random distribution. In addition to the underrepresentation of mutations in genes (which was still significant), we found a significant enrichment of somatic mutations in transposable elements of L1.*

*We have updated the result section accordingly.*

L119: Leaf only mutations: might these be L3?

*Response: We agree that it is a possibility that the leaf-only mutations occurred in L3, but there are indications in the data suggesting that this is not the case.*

*For example, no leaf-only mutations were identified in more than one leaf. If some of these were L3 mutations, we would expect that they were partially shared by multiple leaves from the different branches, similar to what we observed for L1 and L2 mutations.*

*Moreover, our analysis suggested that all the leaf samples are highly enriched for L2 cells. Mutations that are shared in L2 in fruits and leaves have highly similar mutant allele frequencies (orange points in Supplementary Figure S16), which is an indication that leaves samples mostly resemble the DNA extracted from L2. As almost all leaf-only mutations have high allele frequencies (green points in Supplementary Figure S16), we would speculate that most of them reside in L2 cells in leaves and not in L3 cells. Additional support for this comes from Supplementary Figure S15 showing the cross-section of an Apricot leaf. Here, L2 cells outnumber L1 and L3 cells, corroborating that leaves are enriched for L2 cells, and implying that it is improbable to observe high allele frequency mutations from L3. This further highlights the impact of morphology and cell lineage distribution of complex organs on somatic mutation identification.*

Line 175-178: Any more info on mother tree or when this cross might have taken place and how many rounds of clonal propagation since?

*Response: Rojo Pasión was generated through a cross between the North American cultivar ‘Orange Red’ and the Spanish cultivar ‘Currot’ within the apricot breeding program at CEBAS-CSIC in Murcia (Spain) in 1996 (Hough and Bailey, 1982, Egea et al., 2004). Seedlings from this cross were transplanted to fields in the Spring of 1997. In the following four years, the trees were evaluated for Plum Plox Virus tolerance, partial red skin colour, fruit quality and productivity (among other traits), which led to the selection of Rojo Pasión.*

*In 2001, the selected Rojo Pasión tree was grafted onto four rootstock (first round of clonal propagation) to create four new Rojo Pasión trees. In 2009, one of these four trees (unfortunately not clear which one) was used for clonal multiplication resulting in the tree used for this study.*

*With this, we have updated the introduction of the Rojo Pasión tree and added an extra description for the putatively fixed somatic mutations that are shared across all branches as those might be inherited from earlier clonal generations.*

Additional discussion on various results may be useful to readers and increase the impact of the paper.

Line 129: Higher L1 mutation rate. This result is particularly compelling. Perhaps since L1 doesnt contribute to germline, there has been relaxed selection (at long-term evolutionary timescales) on mechanisms promoting DNA repair in L1? Have the authors considered testing for differences in the expression of DNA repair genes between cell layer types using their single-cell RNAseq datasets?

*Response: We agree that variation in DNA repair efficiency could explain higher mutation rates in L1. This hypothesis is supported by an earlier microarray analysis of SAM cells from Arabidopsis thaliana, where cells in L2 were found to have higher expression of DNA repair and telomere maintenance genes (*[*https://doi.org/10.1242/dev.106104)*](https://doi.org/10.1242/dev.106104)*.*

*To test this, we analyzed orthologs of Arabidopsis DNA repair genes in the single cell RNAseq data from the Apricot leaves for expression differences between layers. However, in this data, we could not find significant expression differences. We discuss these aspects in the manuscript.*

It might be worth speculating on consequences for clonal plants/crops/breeding, such as the transgenerational stability of somatic "sports" depending on the cell layer?

*Response: Thank you for the useful suggestion. We have updated the Conclusion section and now discuss the transgenerational aspects of sport mutants in respect to cell layers.*

Line 162: L1 are more similar between branches than with L2 of same branch. This is a main point. Do the authors mean to reference Figure 1J to show this? I wonder if a version of Fig S10 which combines L1 and L2 into a single distance tree would be valuable?

*Response: Again, thank you for the very helpful comment. We totally agree that this is a main point. We have added a new Figure 2 to highlight this point, where we present combined “phylogenetic” analyses for L1 and L2.*

*There are two reasons why layers of distant branches are more similar to each other than the different layers of one branch:*

1) *Propagation of layer-specific mutations during clonal reproduction. During grafting, the layer-specific somatic mutations can be fixed in the grafted branch, in consequence these mutations will then be shared by all branches of the tree of the following generation. This exciting hypothesis is supported by the 22 layer-specific mutations shared across the entire tree.*
2) *Propagation of layer-specific mutations during branching. Within a clonal generation, somatic mutations, which happen in individual layers of the meristem, are propagated during branching. This accumulates mutations in specific layers which results in divergence between layers. This hypothesis is supported by the “phologenetic” analysis of the subtrees.*

*Taken together, these factors suggest that in clonally propagated plants/crops, somatic mutations accumulate with every clonal generation. As a result, the layers will continue to diverge over generations (unless cellular exchanges occur between the layers).*

*Now, we discuss these specific points in more detail in the manuscript.*

L 343: "genome sequencing (scDNA-seq)" is mentioned but it wasnt immediately clear where scDNA-seq appears in results?

*Response: We generated scDNA-seq for four leaves as we were interested in identifying somatic mutations in single cells, however, somatic mutation identification in this single-cell sequencing data was less efficient and we analysed this data in bulk only. We updated the manuscript and now mention this.*

# Reviewer #2

This manuscript describes the distribution of somatic mutations associated with the L1 and L2 lineages in apricot using tissue specific DNA sampling and single-cell RNA sampling. The authors state that they can disregard the contribution of the L3 layer. They report a high number of accumulated mutations in both the L1 and L2 layers, shared across seven fruit sampled from seven major branch units across the tree. This is one of the first studies to assign mutations to specific cell layers in plants. Moreover, it uses two RNA methodologies scRNA seq and SclSO-seq that are among the first applications of such methods to somatic mutation studies. While the study is novel there are also several criticisms that relate to inference regarding the layers of the fruit samples.

*Response: Thank you very much for the positive feedback and pointing out the novelty of our work. We agree that L3 mutations should not be ignored, and we certainly did not intend to disregard their general contribution. However, as L3 cells are only present as a very thin cell layer around the stone in the fruits, it is difficult to extract sufficient amounts of L3 DNA. As a result, we have not analysed L3 mutations in this study. We have updated the manuscript to explain this better.*

Most important of these is the author statement (Lines 101-2) that they can disregard the contribution of the L3 layer. Derman (1956) seems to show clearly through the cytochimeric mutants (Fig. 13) and with the L3 bud sports that the L3 layer is fully intercalated with L2 in the mesocarp of the fruit of peach, which is a closely allied crop. Thus, isn't it possible that some of the SMs in the current study that are classed as L2 mutations could possibly be L3 mutations? Given that all progeny from this tree will all derived from L2, as stated by the authors, then the answer could be determined from the progeny or successive branches in L2 of next years. Alternatively, if progeny data are not available, then the authors could add language to leave the interpretation more open to a contribution of L3. Given that Stewart and Dermen (1975) suggest that L3 is often replaced by L2 along shoots, this could be mentioned to support the emphasis on L2.

*Response: As mentioned above, we did not intend to imply that the effects of L3 can be disregarded and we have updated the text to describe this better.*

*Following your comment, we have added a more open interpretation regarding the origin of what we refer to as L2 mutations. For this, we have updated the conclusion and discussed the possible influence of cellular migration between L2 and L3.*

The other sampling issue relates to the suture line of the fruit which is L1 only, of two cell thickness, and entirely bissects the fruit interior. This presence of L1 in the interior of the fruit is discussed in several figures in the Derman (1956) paper, notably Fig. 15 and Fig. 6D. It was unclear to this reviewer how the mesocarp sampling in the current manuscript addressed the suture region. Was effort made to avoid the suture line and thus possible presence of L1 cells into the collected samples? The reader could not find text addressing this issue in the methods section. Currently, the manuscript reports that twelve mutations are shared by L1 and L2 (Fig. 2B and Fig. 1J far right bar group) and the text interprets that this could be due to layer replacement (Lines 124-5). If layer replacement were actually to occur in the meristem leading to that fruit,would it not have a much more profound effect on the mutation distribution of the whole distal samples? Thus, to this reader, the more likely conclusion is a small amount of L1 cell contamination into the L2 samples from the suture zone.

*Response: Thank you for pointing out the missing description of the suture region. We removed the tissue of the suture regions before extracting DNA from L2 layer because of the reasons that you mentioned. This is now mentioned in the method section.*

*We also agree that imperfect enrichment of cell layers could result in a signal that implies shared mutations. While it is hard to avoid this (or prove the absence of this) in an experimental setting like ours, we have updated the text and mentioned this valuable point as well.*

Figure 3 needs more guidance for the reader. In panel 3B, does Cluster 0 represent the largest group of cells and Cluster 14 represent the smallest group of cells? The label for Cluster six is possibly missing. For panel 3C, the reader is shown the observed hits of cells with each of the six mutations, but the reader cannot tell from the panel or the legend what was expected. Were these six mutations supposed to be present in all four branches? If some of them were only expected in a subset of branches, then it will help to have that information, at least in the figure legend.

*Response: Thank you for the suggestion. We agree that Fig 3c (now Figure 4c) was complicated to understand from the visual alone. Based on your suggestions, we have updated the figure and corresponding legend to highlight the branches in which each mutation was identified.*

*The proportion of cluster sizes is shown in Supplementary Figure S18. While Cluster 0 is in fact the largest cluster in all datasets, the numbering does not follow the cluster size in general, as the ranking of the cluster sizes is different in the different samples.*

*Thank you for pointing out the missing label. It is now added.*

Additional comments:

1. Legend for Fig. 1C could use clarification of the units of the Y-axis.

*Response: Agreed, we have updated the corresponding legend.*

2. Fig. 2C, the number of SMs that were used to determine the percentages shown would help. Not clear to the reader how the two panels differ and how data from specific mutations were used. Are data from all of the mutations shown here or just for a subset of mutations?

*Response: Thank you for the helpful suggestion. We have updated the corresponding figure legend (now Fig. 3C) and now mention these numbers. We have also added a new supplementary methods section that describes the details of the genotyping analysis.*

*For the genotyping analysis, we used point mutations identified in L1 and L2 layers extracted from fruits. We did not use indel mutations as genotyping indel alleles with low read coverage is highly error-prone. We genotyped 50 and 40 mutations found in L1 and L2, respectively. These two sets formed the two panels shown in the figure. Some mutations were identified in multiple branches. For such mutations, we genotyped the layer and leaf samples of every branch in which the mutation was identified. This resulted in 99 and 51 genotyping tests for L1 and L2, respectively (Supplementary Table S3). The percentages shown in Figure 2C (now Figure 3C) were based on the number of genotyping tests.*

3. What is the cutoff in Appendix Table S4 for allele to be considered present in a sample? Not clear to the reader how to understand the quantitative variation shown there.

*Response: The information in Supplementary Table S4 was not used for calling mutations. Somatic mutations were identified using custom pipelines as described in the “Somatic mutation identification using layer-enriched fruit DNA” and the “Somatic mutation identification in leaves” method sections. In brief, we used base quality, mapping quality, sample-specific read-depth, alternate allele frequency, and manual curation to call somatic mutations. These mutations are reported in Supplementary Table S3.*

*In Supplementary Table S4, we reported the alternate allele frequencies at these positions across all samples. Supplementary Table S4 can be used to study the variance in mutant allele frequencies at the mutated sites. For example, from Supplementary Table S3, a mutation at CUR1G:1859794 is reported to be present in layer L1 of branch B6. Supplementary Table S4 shows that the alternate allele frequency of the mutated allele is 0.92 in L1 in branch B6 and 0 everywhere else.*

*We have updated the caption of Supplementary Table S4 to inform the reader on how to understand the table.*

**Reviewer #3**

This manuscript studied somatic mutations in distinct cell layers of fruit samples collected from multiple branches of an Apricot tree. The results showed that >90% of the somatic mutations were specific to individual layers. Overall, the findings of this study are generally interesting, and important for plant breeding research as well.

*Response: Thank you very much for your positive evaluation.*

Although the study is well done, I have a few questions or suggestions.

1) To me, it is difficult to imagine the different layers of the tree across a big tree evolved like this study: different layer has different mutation rate, and the same layer share more mutations among different branches. It is important to give some explanation based on the references or proposal some assumptions about this.

*Response: Angiosperms grow from meristems, which are highly structured organs of stem cells at the tip of developing tissue. They proliferate into axillary meristems or organs that are formed successively. Of course, this is not a new observation but has been studied in many different plant lineages for a long time. Among others, pivotal work on this was done by Dremen and others as mentioned by Reviewer 2. The first few references of the manuscript describe all the different aspects of this.*

*In this study, we have integrated cell lineages, a consequence of the layer structure of the meristems, into the analysis of somatic mutations. For this, we hypothesised that somatic mutations are mostly restricted to the cell lineages in which they occurred. This intuitive hypothesis has not been considered in somatic mutation analysis studies before.*

2) Initially, when I read the manuscript, I assumed different branches mimics mutation accumulation lines, it will be important to discuss the similarity and difference between MA study and this study.

*Response: Mutation accumulation (MA) lines are generated through sexual reproduction across generations. Each generation includes the formation of a zygote and thus the fixation of somatic mutation (which then turns into germline mutations).*

*Somatic mutations occur in individual somatic cells and proliferate to subsequent daughter cells within the same organism. Whether such cell lineages mimic MA lines, including the effects of selection, is still unclear. However, in this study, we addressed the special distribution of somatic mutations across the different branches of a tree that resulted in the mosaicism we described.*

3) The reference genome used here are assembled with the reads produced from leaves of the seven different branches. I would assume the assembled genome must already contain most of the mutation of different branches, which is kindly a mixed genome. To what extent this mixed genome could affect variation identification and final results?

*Response: The DNA for the reference genome assembly was extracted from leaves collected from multiple branches. While we agree that this sequence is a mosaic of different alleles, the identification of mutations is not dependent on the reference alleles. Somatic mutations are identified by comparing samples to each other rather than to the reference sequence.*

*Consequently, whether the reference sequence supports the wild-type or the mutant allele, somatic mutation identification would not be biased.*

*Therefore, we do not expect the assembly to introduce biases in our variation identification pipeline or the final results.*

**Second round of review**

**Reviewer 1**

The authors have addressed all of my comments. I am excited to see this work published!

**Reviewer 2**

This version has excellent improvements to the figures and text. It is a novel and interesting contribution and is well suited to the journal. The authors satisfactorially addressed all of my comments.

**Reviewer 3**

The revision addressed all my concerns, I am satisfied with the revision.
